# Insulin resistance is associated with epigenetic and genetic regulation of mitochondrial DNA in obese humans

**DOI:** 10.1186/s13148-015-0093-1

**Published:** 2015-06-10

**Authors:** Louise D. Zheng, Leah E. Linarelli, Longhua Liu, Sarah S. Wall, Mark H. Greenawald, Richard W. Seidel, Paul A. Estabrooks, Fabio A. Almeida, Zhiyong Cheng

**Affiliations:** Department of Human Nutrition, Foods and Exercise, Fralin Translational Obesity Research Center, College of Agriculture and Life Science, Virginia Tech, Blacksburg, Virginia USA; Department of Family and Community Medicine, Carilion Clinic, Roanoke, Virginia, USA; Department of Psychiatry, Carilion Clinic, Roanoke, Virginia, USA

**Keywords:** DNA methylation, D-loop, Mitochondrial regulation, Metabolism, Obesity, Insulin resistance, Genetic, Epigenetic

## Abstract

**Background:**

Mitochondrial alterations have been observed in subjects with metabolic disorders such as obesity and diabetes. Studies on animal models and cell cultures suggest aberrant glucose and lipid levels, and impaired insulin signaling might lead to mitochondrial changes. However, the molecular mechanism underlying mitochondrial aberrance remains largely unexplored in human subjects.

**Results:**

Here we show that the mitochondrial DNA copy number (mtDNAn) was significantly reduced (6.9-fold lower, *p* < 0.001) in the leukocytes from obese humans (BMI >30). The reduction of mtDNAn was strongly associated with insulin resistance (HOMA-IR: −0.703, *p* < 0.05; fasting insulin level: −0.015, *p* < 0.05); by contrast, the correlation between fasting glucose or lipid levels and mtDNAn was not significant. Epigenetic study of the displacement loop (D-loop) region of mitochondrial genome, which controls the replication and transcription of the mitochondrial DNA as well as organization of the mitochondrial nucleoid, revealed a dramatic increase of DNA methylation in obese (5.2-fold higher vs. lean subjects, *p* < 0.05) and insulin-resistant (4.6-fold higher vs. insulin-sensitive subjects, *p* < 0.05) individuals.

**Conclusions:**

The reduction of mtDNAn in obese human subjects is associated with insulin resistance and may arise from increased D-loop methylation, suggesting an insulin signaling-epigenetic-genetic axis in mitochondrial regulation.

**Electronic supplementary material:**

The online version of this article (doi:10.1186/s13148-015-0093-1) contains supplementary material, which is available to authorized users.

## Background

The epidemic of obesity is growing globally. In addition to the changes in glucose and lipid metabolism, obesity is associated with insulin resistance and increased risk of type 2 diabetes (T2D) and cardiovascular diseases (CVD) [1–3]. As the primary metabolic platform in mammalian cells, mitochondria undergo genetic and epigenetic regulation, which leads to alterations in mitochondrial function, dynamics, and biogenesis during metabolic disorders [2, 4, 5]. Decrease in mitochondrial DNA copy number (mtDNAn) has been observed in skeletal muscle, adipose tissue, and peripheral blood from obese and T2D individuals [6–15]. The reduced mtDNAn in peripheral blood was found to precede the development of T2D [10, 16]. In addition, DNA methyltransferase 1 (DNMT1) can translocate to the mitochondria and catalyze mitochondrial DNA (mtDNA) methylation, thereby manipulating the expression of transcripts from the heavy and light strands of mtDNA [17]. It was shown that, in the elderly or individuals with insulin resistance and T2D, mitochondrial COX7A1 (the subunit of cytochrome c oxidase or complex IV in the respiratory chain) and NDUFB6 (subunit in complex I in the respiratory chain) were dysregulated, concomitant with higher DNA methylation in the promoters of COX7A1 and NDUFB6 [18, 19]. Recently, Pirola et al. found that the methylation of MT-ND6 (mitochondrial NADH dehydrogenase) was higher in the liver of nonalcoholic steatohepatitis (NASH) than simple steatosis patients, and MT-ND6 methylation inversely correlated with MT-ND6 transcription and protein expression in the liver of NASH patients [20]. The change in MT-ND6 methylation was significantly associated with nonalcoholic fatty liver-disease activity score [20]. These findings suggest that changes in mtDNAn and mtDNA methylation may play an important role in metabolic disorders.

Mitochondrial alteration reflects metabolic status. The genes and proteins controlling mitochondrial dynamics can be dysregulated by high glucose, leading to overproduction of reactive oxygen species and insulin resistance [21–26]. The evidence from genetically modified mice suggested that overloading mitochondria by lipids led to incomplete fatty acid oxidation, mitochondrial stress, and impaired insulin signaling [27, 28]. A feedback loop was recently discovered, showing that insulin resistance results in mitochondrial changes in cell and animal models, as well as in human subjects [29–32]. However, it is not well defined how the metabolic changes might be related to genetic and epigenetic regulation of mitochondria. In this study, we recruited obese and lean subjects to investigate the mtDNAn and DNA methylation in the displacement loop (D-loop) region of the mitochondrial genome, which controls the replication of mtDNA and organization of the mitochondrial nucleoid [33–35]. We detected a significantly increased DNA methylation in the D-loop region, which was concomitant with decreased mtDNAn in the obese individuals when they were compared with the lean subjects. Moreover, the change in mtDNAn was strongly associated with insulin resistance, but not with impaired fasting glucose or dyslipidemia (e.g., triglyceride, cholesterol, and VLDL). Our study provides new evidence critical for the ongoing journey in discovering mtDNA methylation and exploring its role in metabolic regulation [18–20, 36] and suggests an insulin signaling-epigenetic-genetic axis that may control mitochondrial regulation.

## Results

### Metabolic changes in obese subjects

Among the 40 participants, 32 people had a BMI greater than 30 (mean value = 36.6; referred to later as obese group) and 8 showed BMI below 25 (mean value = 23.1; referred to later as lean group), with the difference between the two groups being significant (*p* < 0.0001). As shown in Table 1 and Additional file 1: Figure S1, the obese group showed a significant impairment in fasting glucose (95.9 ± 2.4 vs. 83.9 ± 1.9 in the lean group, *p* < 0.05), and fasting insulin levels dramatically increased (21.8 ± 2.5 vs. 9.4 ± 1.6 in the lean group, *p* < 0.05), suggestive of impaired insulin sensitivity or development of insulin resistance [37, 38]. Insulin resistance was further confirmed by the HOMA-IR value, which was 2.7-fold (*p* < 0.05) higher in the obese group than in the lean one. Moreover, the plasma LDL level (115.6 ± 4.8 vs. 94.4 ± 8.3, *p* < 0.05), low-density lipoprotein (LDL)/high-density lipoprotein (HDL) ratio (2.2 ± 0.1 vs. 1.7 ± 0.2, *p* < 0.05), and total cholesterol/HDL ratio (3.8 ± 0.2 vs. 3.0 ± 0.3, *p* < 0.05) all showed significant elevation. These findings suggest that the obese group had impairment in insulin signaling, concurrent with aberrant glucose and lipid metabolism.

**Table 1:** Demographic and metabolic characteristics of participants

Characteristics	Lean (*n* = 8)	Obese (*n* = 32)
Sex (male/female)	2/6	8/24
Age (years)	28.1 ± 4.5	49.5 ± 2.4**
BMI (kg/m^2^)	23.1 ± 0.6	36.6 ± 1.2***
Fasting glucose (mg/dL)	83.9 ± 1.9	95.9 ± 2.4*
Fasting insulin (μU/mL)	9.4 ± 1.6	21.8 ± 2.5*
HOMA-IR	1.94 ± 0.32	5.31 ± 0.68*
Triglyceride (mg/dL)	94.9 ± 16.6	145.8 ± 20.5
HDL (mg/dL)	59.8 ± 4.1	53.8 ± 2.1
LDL (mg/dL)	94.4 ± 8.3	115.6 ± 4.8*
VLDL (mg/dL)	19.0 ± 3.3	29.2 ± 4.1
LDL/HDL ratio	1.7 ± 0.2	2.2 ± 0.1*
Total cholesterol (mg/dL)	173.2 ± 7.5	195.2 ± 6.2
Total cholesterol/HDL	3.0 ± 0.3	3.8 ± 0.2*
HbA1c (%)	5.4 ± 0.1	5.7 ± 0.1

Mean ± SE; *p < 0.05; **p < 0.001; ***p < 0.0001

### mtDNAn was reduced in obese subjects

**Fig. 1:**
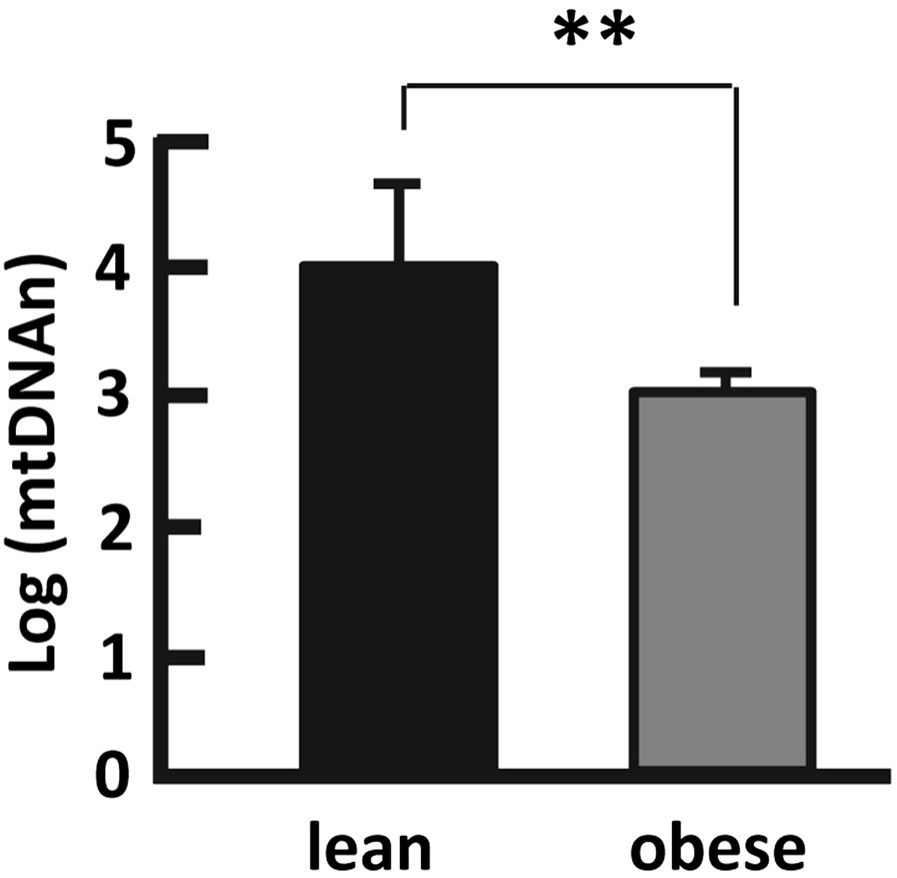
Measurement of mitochondrial DNA copy number (mtDNAn) in lean (BMI <25 kg/m^2^) and obese (BMI >30 kg/m^2^) subjects. *n* = 8–32; **p < 0.001

The mitochondrial genome or mtDNA encodes 13 protein components of the respiration chain that underpin mitochondrial function [39, 40]. We found that the mtDNAn in the obese group was 6.9-fold lower (delta log-mtDNAn = 0.84, *p* < 0.001) when compared with their lean counterparts (Fig. 1). Given the significant age difference shown in Table 1 and Additional file 1: Figure S1, we conducted an age-matched analysis of mtDNAn, which indicated an mtDNAn tenfold lower (delta log-mtDNAn = 0.99, *p* < 0.05) in obese the group than in the lean group (Additional file 2: Figure S2). This is consistent with a previous report showing lower mitochondrial content in the skeletal muscle and adipose tissues from obese individuals [7–9, 11]. Because changes in mtDNAn can affect the integrity, assembly, and operation of the mitochondrial respiratory chain [41, 42], it is conceivable that the mitochondrial function or capacity is impaired in obese subjects.

### Alteration of mtDNAn was associated with insulin resistance

**Fig. 2:**
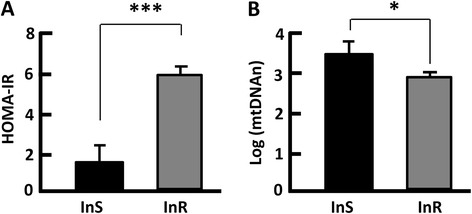
Measurement of mtDNAn in insulin-sensitive (InS) and insulin-resistant (InR) individuals. **a** HOMA-IR indicates InS and InR groups, with the cutoff point set at 2.5. **b** The mtDNAn was significantly lower in InR individuals than InS subjects. *n* = 13–27; *p < 0.05; ***p < 0.0001

To examine how mtDNAn alteration was associated with the metabolic changes in obese subjects, we compared the mtDNAn in the insulin-sensitive (InS) group with that of the insulin-resistant (InR) group by setting the cutoff point at 2.5 for HOMA-IR [43, 44]. The InR group had a mean value of HOMA-IR that was 3.8-fold higher than the InS group (*p* < 0.0001), indicative of impaired insulin action [43, 44]. However, the mtDNAn in the InR group was 3.2-fold lower (delta log-mtDNAn = 0.5, *p* < 0.05) in comparison with the InS group (Fig. 2). These findings support the notion that insulin resistance links mitochondrial alteration to metabolic disorder [29–32, 45].

### Alteration of mtDNAn was independent from aberrant glucose and lipid levels

**Fig. 3:**
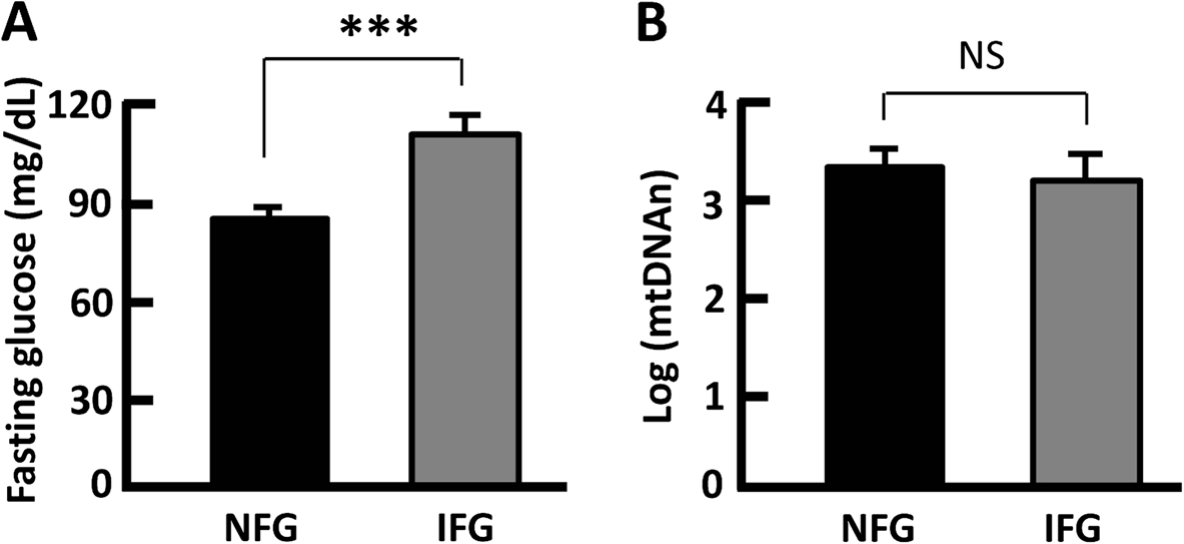
Measurement of mtDNAn in normal fasting glucose (NFG) and impaired fasting glucose (IFG) individuals. **a** Fasting glucose levels in NFG and IFG groups, with the cutoff point set at 100 mg/dL. **b** The mtDNAn in IFG individuals was comparable to that of NFG subjects. *n* = 11–29; ***p < 0.0001; NS, not significant

**Fig. 4:**
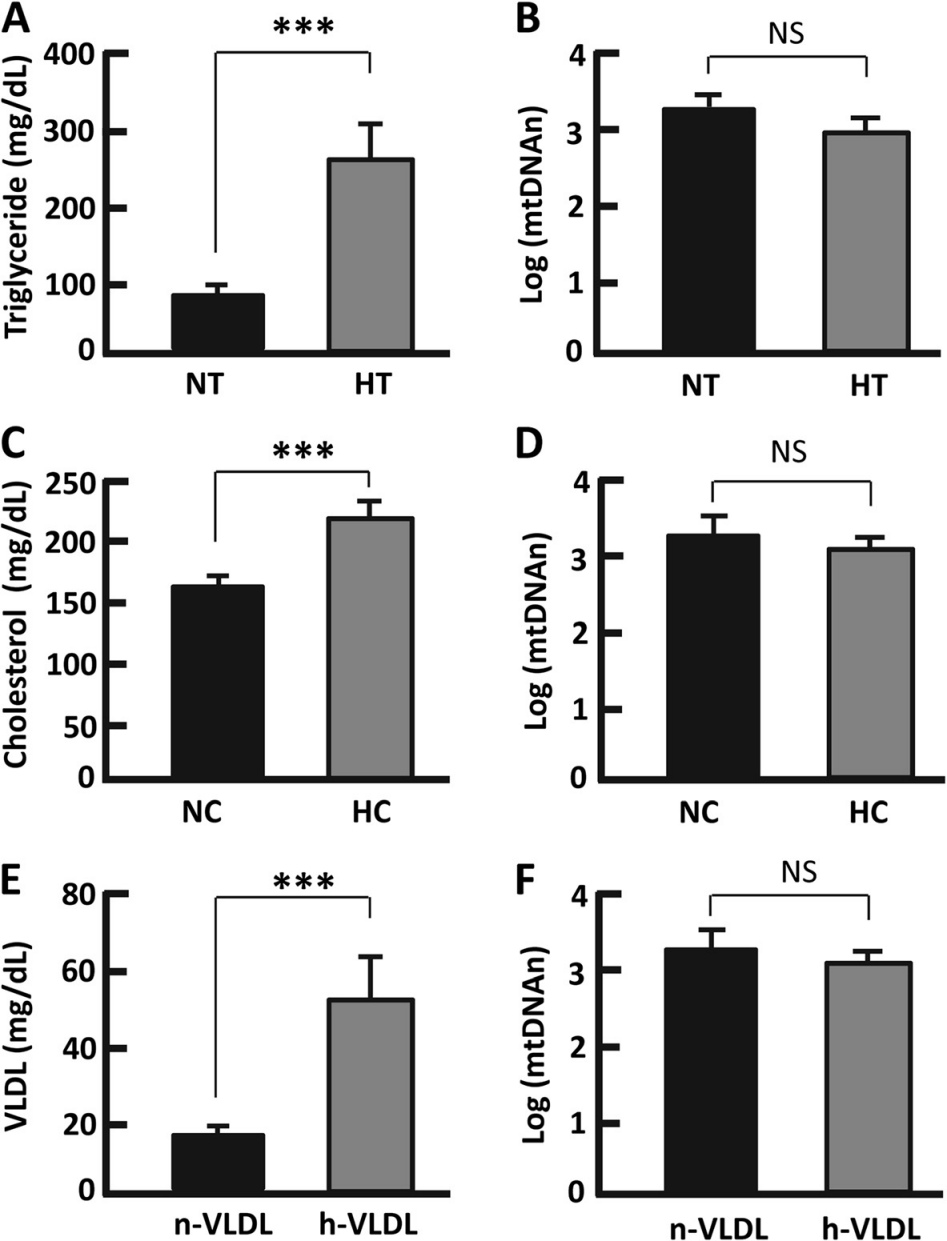
Measurement of mtDNAn in individuals with normal lipid levels and dyslipidemia. **a**, **b** the fasting plasma triglyceride (**a**) and mtDNAn (**b**) in individuals with normal triglyceride (NT) and high triglyceride (HT), with the cutoff point set at 150 mg/dL; *n* = 10–30. **c, d** the fasting plasma cholesterol (**c**) and mtDNAn (**d**) in individuals with normal cholesterol (NC) and high cholesterol (HC), with the cutoff point set at 200 mg/dL; *n* = 12–28. **e, f** the fasting plasma VLDL (**e**) and mtDNAn (**f**) in individuals with normal VLDL (n-VLDL) and high VLDL (h-VLDL), with the cutoff point set at 32 mg/dL; *n* = 9–31. ***p < 0.0001; NS, not significant

According to the American Diabetes Association (ADA), a value of less than 100 mg/dL is defined as normal fasting glucose (NFG), while a value greater than 100 but less than 125 mg/DL indicates impaired fasting glucose (IFG) [46]. In the IFG group, the fasting glucose level was 111 mg/dL on average, significantly higher than that of the NFG group (86 mg/dL on average, *p* < 0.0001). However, the mtDNAn values of these two groups showed no significant difference (Fig. 3). In addition, mtDNAn did not change with plasma lipid levels, irrespective of the aberrantly higher concentrations of plasma triglyceride, cholesterol, LDL, and VLDL (Fig. 4 and data not shown) [47]. Together, these results suggest that mtDNA alteration may arise from insulin resistance rather than aberrant glucose and lipid levels (Figs. 2, 3, and 4).

### Regression analysis suggested an insulin signaling-mtDNAn axis

To further validate the relationship between mtDNAn and the metabolic parameters, we conducted regression analysis, and the results were shown in Table 2 and Additional file 3: Figure S3. Consistent with the above observation (Figs. 1, 2, 3, and 4), mtDNAn showed a negative correlation with BMI (−0.026; *p* < 0.05) and with HOMA-IR (−0.703, *p* < 0.05). Because fasting insulin levels can also indicate insulin resistance to some extent as HOMA-IR does, it was negatively correlated with mtDNAn (−0.015, *p* < 0.05) [37, 38]. By contrast, mtDNAn did not have a significant correlation with fasting glucose or lipid levels (Table 2 and Additional file 3: Figure S3). Additionally, age-dependent decrease of mtDNAn was not significant (Table 2 and Additional file 2: Figure S2), in line with the previously observed lack of mtDNAn change with age in human skeletal muscle and myocardium [41]. A recent study suggested that mtDNAn alteration in peripheral blood cells did not initialize until the age of 50 years [48], which may account for the lack of significant correlation between mtDNAn and age in this study as the majority of our participants were younger than 50 years. Together, our results suggest an insulin signaling-mtDNAn axis in human leukocytes (Fig. 2, Table 2, and Additional file 3: Figure S3).

**Table 2:** Univariate regression analysis of mtDNAn among all subjects

Parameters	β coefficient	*R* value	*p* value
Age	−0.008	0.212	0.189
BMI	−0.026	0.333	*0.041*
Fasting glucose	−0.004	0.084	0.604
Fasting insulin	−0.015	0.320	*0.044*
HOMA-IR	−0.703	0.379	*0.016*
Triglyceride	−0.001	0.163	0.314
HDL	0.010	0.187	0.248
LDL	−0.002	0.095	0.569
VLDL	−0.005	0.165	0.310
LDL/HDL ratio	−0.130	0.133	0.425
Total cholesterol	−0.001	0.050	0.760
Cholesterol/HDL	−0.116	0.178	0.272
HbA1c	0.182	0.115	0.480

### D-loop had higher methylation in obese subjects

**Fig. 5:**
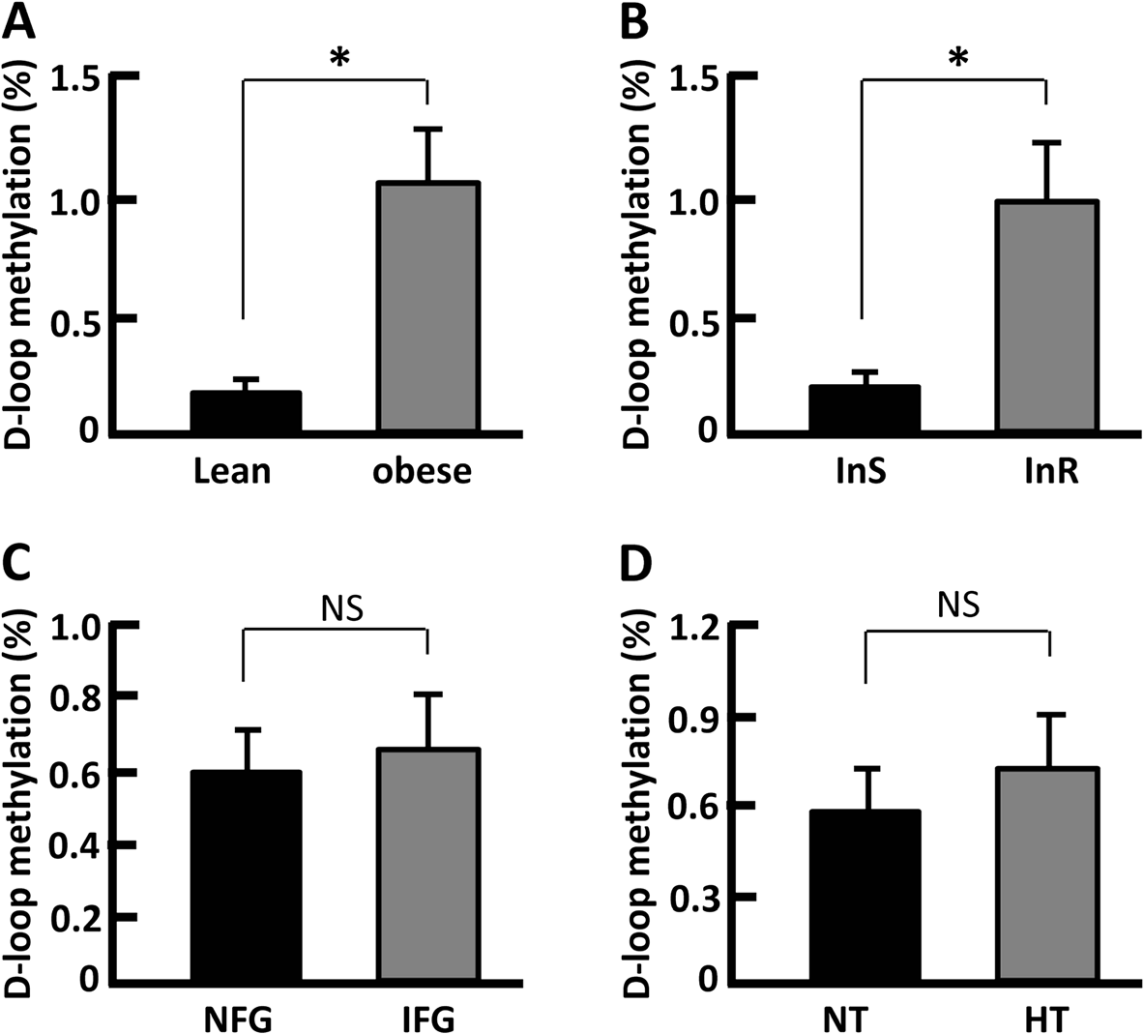
Measurement of DNA methylation in the D-loop region of mitochondrial genome. **a** D-loop methylation in lean and obese subjects; *n* = 8–32. **b** D-loop methylation in InS and InR subjects; *n* = 13–27. **c** D-loop methylation in NFG and IFG individuals; *n* = 11–29. **d** D-loop methylation in NT and HT individuals; *n* = 10–30. The DNA methylation levels were also compared between NC and HC, n-VLDL, and h-VLDL groups, and there was no significant difference (not shown). *p < 0.05; NS, not significant

The D-loop region controls the replication of the mitochondrial DNA and organization of the mitochondrial nucleoid [33–35]. The observation of reduced mtDNAn in the obese individuals (Figs. 1 and 2) prompted us to ask whether the D-loop region underwent aberrant methylation, the modification that may regulate mtDNA replication and transcription [49]. Regression analysis suggested that mtDNAn was negatively correlated with D-loop methylation (−0.078; *p* < 0.05). Moreover, DNA methylation in the D-loop region was 5.2-fold higher in the obese group than in the lean group (*p* < 0.05, Fig. 5a). Interestingly, the increased methylation of D-loop was phenocopied by insulin-resistant (InR) subjects, and DNA methylation was 4.6-fold higher than that of insulin-sensitive (InS) subjects (*p* < 0.05, Fig. 5b). However, the difference of D-loop methylation was indiscernible between the NFG and IFG groups (Fig. 5c) or between normal triglyceride (NT) and hypertriglyceridemic (HT) groups (Fig. 5d). Therefore, the increased DNA methylation in the D-loop region was associated with insulin resistance but independent from aberrant glucose and lipid levels. Our data adds new and timely evidence to the potential role of mtDNA methylation in metabolic regulation [18–20, 36].

## Discussion

The growing epidemic of obesity is largely attributed to the current life style of energy overconsumption with inadequate physical activity [2, 50, 51]. As such, the surplus of nutrients is accumulated and contributes to the interactions between genes and environment [2, 51]. Mitochondrial alterations have been observed in obese individuals, including impaired mitochondrial fatty acid/lipid oxidation capacity in the skeletal muscle and reduced mtDNAn in adipose tissues and peripheral blood samples [6–9, 11]. However, whether an epigenetic mechanism underlies the reduced mtDNAn has not been defined, and how genetic and epigenetic traits in mitochondria are related to altered metabolic parameters remains elusive. In this study, we found that the reduction of mtDNAn was associated with increased DNA methylation in the D-loop, the critical region that controls the replication of mtDNA, transcription and organization of the mitochondrial nucleoid (Figs. 1, 2, and 5) [33–35, 49, 52, 53]. Moreover, mitochondrial genetic and epigenetic changes seem to be independent from impaired fasting glucose and dyslipidemia but have strong correlation with insulin resistance (Figs. 1, 2, 3, 4, 5, and Table 2). Our results suggest an insulin signaling-epigenetics-genetics axis in mitochondrial regulation. Given the ongoing debate on mtDNA methylation in the literature [36], our study provides new and timely evidence that paves the avenue to understanding metabolic changes in the view of mitochondrial epigenetics [18–20].

Mitochondria have an independent circular genome of 16.5 kb in humans, encoding 13 proteins that assemble the electron transport chain and ATP synthase [39, 40]. Normal mtDNAn and the integrity of the mtDNA molecule account for a functional mitochondrial genome,and are critical for assembly and operation of the respiratory chain [41, 42]. In the obese and insulin-resistant individuals, mtDNAn was significantly reduced and concomitant with the elevation of DNA methylation in the D-loop region, the event that may suppress mitochondrial transcripts and assembly of the respiration chain (Figs. 1, 2, and 5) [2, 53, 54]. While further study is warranted to define how insulin resistance may directly induce the epigenetic and genetic changes, we envision that the recently identified mitochondrial DNMT1 may be an important player with the nicotinamide adenine dinucleotide (oxidized form) (NAD^+^)-dependent deacetylase SIRT1 [17, 29, 55]. It was shown that DNMT1 could be de-acetylated by SIRT1 in a NAD^+^-dependent way, thereby manipulating DNMT1 activity in regulating gene expression [56–58]. In insulin-resistant patients, the gene and protein levels of SIRT1 in peripheral blood cells were significantly reduced, while the expression of other sirtuin family members (SIRT2-SIRT7) was normal in comparison to insulin-sensitive individuals [55]. Moreover, our previous study demonstrated that insulin resistance could reduce cellular NAD^+^ levels and SIRT1 activity in vivo [29]. Thus, we propose that insulin resistance may regulate DNMT1 activity and DNA methylation in the D-loop region through NAD^+^-SIRT1, and this mechanism should be further explored in future studies.

Although aberrant lipid and glucose loads were previously shown to induce mitochondrial changes in cell cultures and animal models [23, 28], we did not observe a significant correlation between altered mtDNAn (or D-loop methylation) and fasting glucose or lipid levels (Figs. 3, 4, and Table 2), presumably because the changes in glucose and lipids were moderate (e.g., the impaired fasting glucose was 95.9 ± 2.4 mg/dL) or because the changes were still in a neonatal stage given that the timing and duration affect metabolic and mitochondrial phenotype [10, 59]. Regardless, insulin resistance shows strong association with altered D-loop methylation and mtDNAn (Fig. 2, Fig. 5, and Table 2). In fact, insulin can directly stimulate mitochondrial protein synthesis and promote mitochondrial function in healthy people, but these effects were absent in insulin-resistant subjects [60, 61]. These findings, along with our discovery of the insulin signaling-epigenetic-genetic axis in this study, strongly suggest that the primary link between insulin signaling and mitochondria is critical for normal metabolism. To this end, use of insulin sensitizers (e.g., pioglitazone and rosiglitazone) has been shown to increase mtDNAn and improve metabolic homeostasis [12, 62].

## Conclusions

In summary, our present study reveals for the first time an insulin signaling-epigenetic-genetic axis that may regulate mitochondria. Particularly, our data adds new and timely evidence to the emerging role of mtDNA methylation in metabolic regulation, paving the avenue to understanding metabolic disorders from a mitochondrial epigenetics perspective [18–20, 36]. Because this was a sub-study of a larger diabetes-prevention trial (diaBEAT-it trial), we were able to access only a limited amount of samples from the participants, not enabling us to conduct an in-depth study of the regulatory mechanism. However, SIRT1-DNMT1 cascade could play an important role because previous studies showed that only SIRT1 of the sirtuin family (SIRT1-SIRT7) underwent dysregulation in peripheral blood cells from insulin-resistant patients [55] and that SIRT1 directly interacted with DNMT1 and regulated its activity in different cell types [56–58]. Our future study will further establish this epigenetic-genetic regulatory axis, so that novel mechanistic support and guidelines may be provided for lifestyle interventions (e.g., physical activity) through enhancing insulin sensitivity and SIRT1 activity [63, 64].

## Methods

### Subjects

We recruited 40 participants previously enrolled in a larger diabetes-prevention trial (diaBEAT-it trial), with diagnosis of no diabetes or cardiovascular disease [65]. All participants were consented by trained research staff and provided with a copy of their signed informed consent. Participants completed an intake questionnaire which included questions about medical history, current medications, and current health behaviors (e.g., physical activity and dietary behaviors). Additionally, resting blood-pressure measurements were recorded for all participants following standard protocols. All procedures were conducted in accordance with NIH Guidelines and approved by Institutional Review Boards at Carillion Clinic and at Virginia Tech.

### Human experimental protocol

Body composition was determined by trained research staff via a dual-energy X-ray absorptiometry scan at the time of consent. An appointment for the blood draw was scheduled for each participant, and participants were instructed to fast overnight (10–12 h) before their scheduled blood draw at Solstas Labs facility (Roanoke, Virginia). Fasting venous blood samples were collected to determine biochemical indexes, including blood-lipid profile (triglyceride, total cholesterol, HDL-cholesterol, and LDL-cholesterol), fasting plasma glucose, HbA1c,and fasting plasma insulin. The homeostasis model assessment for insulin resistance (HOMA-IR) index was calculated as [fasting insulin (μU/ml) × fasting glucose (mg/dL)/405], as previously reported with minor modification due to different units used [10, 43]. Additional fasting blood was collected in EDTA tubes and was processed immediately to prepare white-blood cells (buffy coat), which were stored at −80 °C until further processing [66, 67].

### DNA extraction and bisulfite treatment

DNA was isolated from the buffy coat using the QIAamp DNA Blood Mini Kit (Qiagen, Hilden, Germany) by following the manufacturer’s instructions. EpiTect Bisulfite Kit (Qiagen) was used for bisulfite conversion and cleanup of DNA, during which unmethylated cytosines were converted to uracils and the methylated cytosines were conserved [20, 68]. DNA quality and quantity were examined with a Synergy H4 Hybrid Multi-Mode Microplate Reader (BioTek Instruments, Winooski, VT, USA) and then stored in aliquots at −20 °C until further assay.

### Measurement of mtDNAn

Mitochondrial DNA copy number (mtDNAn) was measured as previously described [10, 41]. Briefly, 40 ng total DNA was used for real-time PCR with the iQ™ SYBR® Green Supermix (Bio-Rad Laboratories, Hercules, CA, USA) on a ViiA™ 7 Real-Time PCR System (Life Technology, Grand Island, NY, USA). The primers used in this study were 5′-CCAACATCTCCGCATGA TGAAAC-3′ (forward) and 5′-TGAGTAGCCTCCTCAGATTC-3′ (reverse) for CYT-B (mtDNA); 5′-GTTACTGCCCTGTGGGGCAA-3′ (forward) and 5′-CAAAGGTGCCCTT GAGGTT-3′ (reverse) for β-globin (nuclear DNA). The amplicon lengths were 434 bp and 356 bp for CYT-B and β-globin, respectively.

### Measurement of D-loop methylation

The methylation of D-loop region was determined by methylation-specific PCR as descried previously [20, 68]. Briefly, the D-loop sequence 16024–576 (1,122 bp) of the Homo sapiens mitochondrion genome (gi|251831106:c576-1, c16569-16024) was used to identify the CpG island (426–576) and design primers for PCR analysis. The following two pairs of primers were designed: one pair was specific for bisulfite-modified methylated DNA, and the other pair was specific for bisulfite-modified unmethylated DNA amplifying heavy strand. The primers used in this study were TAGGAATTAAAGATAGATATTGCGA (forward, starting position at 434 nt) and 5′-ACTCTCCA TACATTTAATATTTTCGTC-3′ (reverse, starting position at 539 nt) for methylated D-loop; 5′-GGTAGGAATTAAA GATAGATATTGTGA-3′ (forward, starting position at 432 nt) and 5′-ACTCTCCATACATTTAATATTTTCATC-3′ (reverse, starting position at 539 nt) for unmethylated D-loop. The bisulfite-modified DNA was used as a template for methylation-specific PCR (MSP) on a ViiA™ 7 Real-Time PCR System, using SYBR® Green PCR Master Mix (Life Technology, Grand Island, NY, USA). Two MSPs were performed simultaneously to detect the methylated (amplicon size; 106 bp) and unmethylated (amplicon size; 108 bp) D-loop for each sample. The percentage of methylated DNA is calculated as described previously [20, 68].

### Statistics

The data are expressed as the mean ± SE unless otherwise specified. Logarithm-transformed data were used for the analysis of skewed variables, such as HOMA-IR and mtDNAn. Pearson’s correlation and regression analysis was applied to evaluate the relationships among mtDNAn and the metabolic indexes. Statistical significance was set at a probability level of *p* < 0.05.

## Additional files

Additional file 1: Figure S1. Scatter plot of the measurements and demographic characteristics of lean (*n* = 8) and obese (*n* = 32) participants in this study. The middle lines indicate the mean values, and the other two shorter lines indicate SE **p* < 0.05; ***p* < 0.001; ****p* < 0.0001. Additional file 2: Figure S2. Age-matched analysis of mtDNAn in lean (*n* = 7) and obese (*n* = 8) participants. (A) No significant difference existed between the ages of lean (*n* = 7) and obese (*n* = 8) participants. (B) Comparison of mtDNAn between lean (*n* = 7) and obese (*n* = 8) participants. The data were presented as mean ± SE. **p* < 0.05; NS, not significant. Additional file 3: Figure S3. Regression analyses of mtDNAn with metabolic parameters and demographic characteristics (*n* = 40). These graphs correspond to the analysis and data shown in Table 2.

## Abbreviations

BMI: body mass index
COX7A1: the subunit of cytochrome c oxidase or complex IV in the respiratory chain
CVD: cardiovascular diseases
D-loop: displacement loop
DNMT1: DNA methyltransferase 1
HC: high cholesterol
HDL: high-density lipoprotein
HOMA-IR: the homeostatic model assessment (HOMA)-insulin resistance index
HT: high triglyceride
h-VLDL: high VLDL
IFG: indicates impaired fasting glucose
InR: insulin resistant
InS: insulin sensitive
LDL: low-density lipoprotein
mtDNA: mitochondrial DNA
mtDNAn: mitochondrial DNA copy number
NAD^+^: nicotinamide adenine dinucleotide (oxidized form)
NC: normal cholesterol
NDUFB6: subunit in complex I in the respiratory chain
NFG: normal fasting glucose
NT: normal triglyceride
n-VLDL: normal VLDL
SIRT1: sirtuin (silent mating type information regulation 2 homolog) 1
T2D: type 2 diabetes
VLDL: very low-density lipoprotein
