# Supplementary figures and images for: Insulin resistance is associated with epigenetic and genetic regulation of mitochondrial DNA in obese humans
Source: Clin Epigenetics. 2015 Jun 10;7(1):60. doi: 10.1186/s13148-015-0093-1 (PMC4479353; doi:10.1186/s13148-015-0093-1)

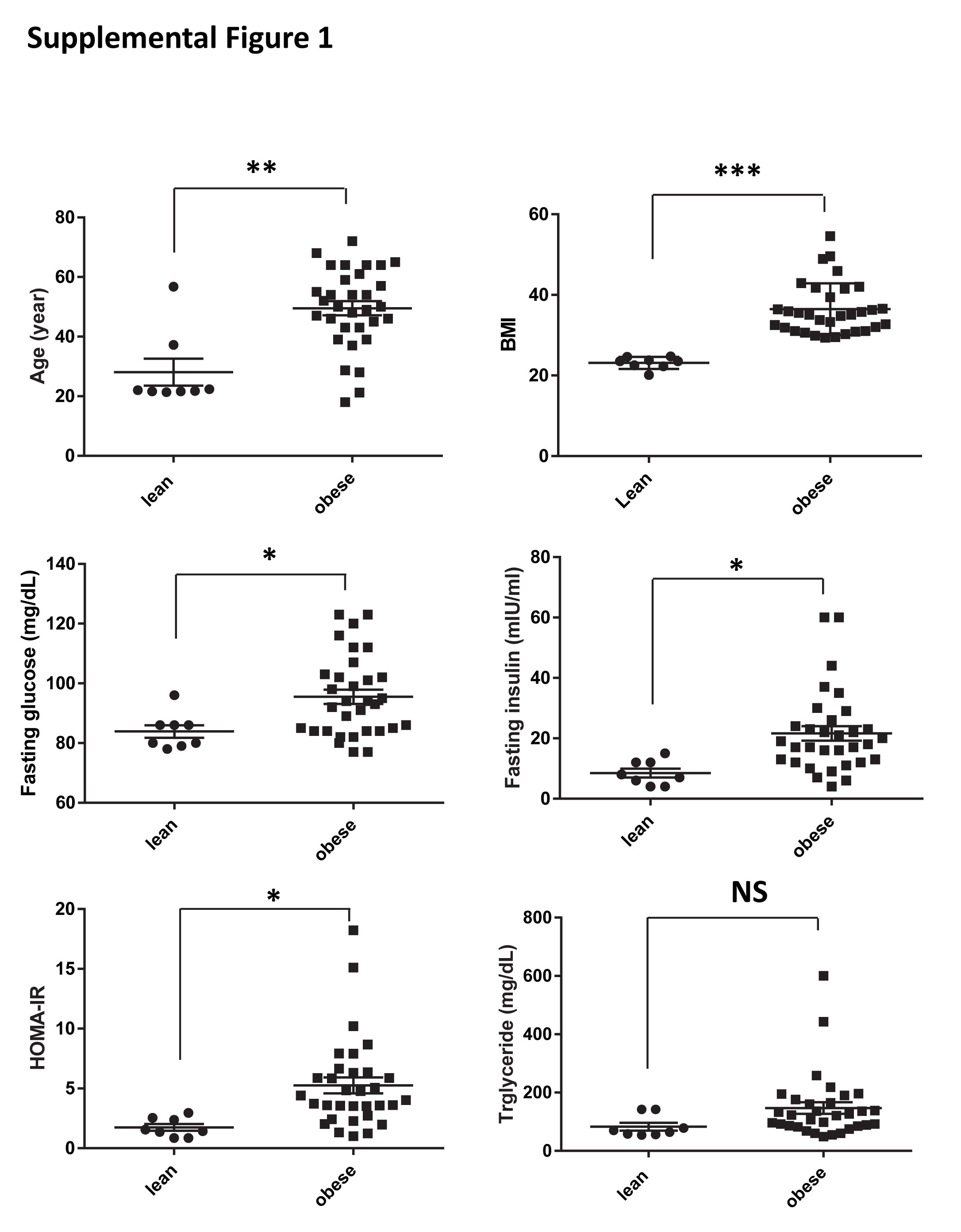

Supplement: Additional file 1: Figure S1. — Scatter plot of the measurements and demographic characteristics of lean (n = 8) and obese (n = 32) participants in this study. The middle lines indicate the mean values, and the other two shorter lines indicate SE *p < 0.05; **p < 0.001; ***p < 0.0001. [file 13148_2015_93_MOESM1_ESM.zip › 13148_2015_93_add1a.jpeg]

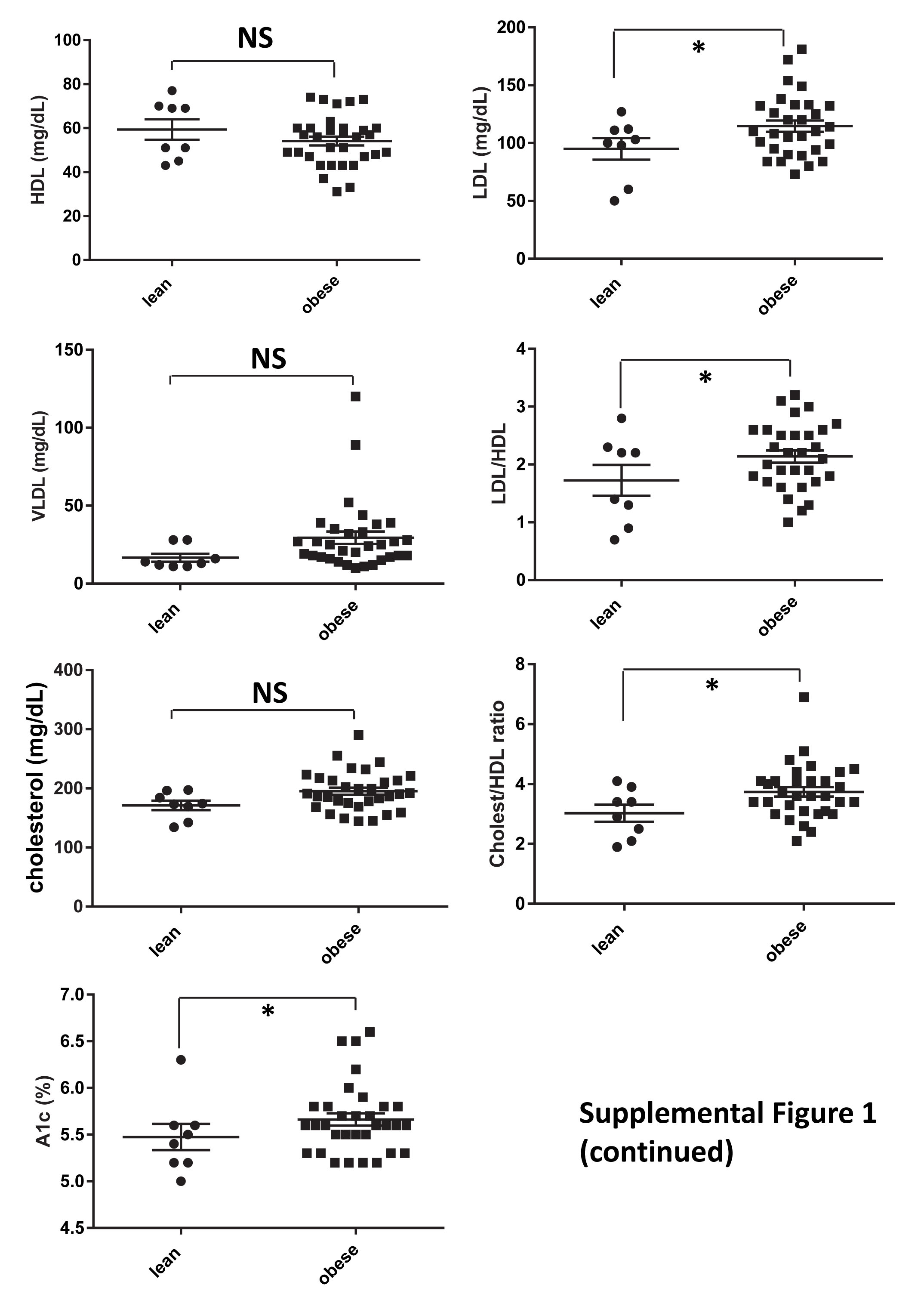

Supplement: Additional file 1: Figure S1. — Scatter plot of the measurements and demographic characteristics of lean (n = 8) and obese (n = 32) participants in this study. The middle lines indicate the mean values, and the other two shorter lines indicate SE *p < 0.05; **p < 0.001; ***p < 0.0001. [file 13148_2015_93_MOESM1_ESM.zip › 13148_2015_93_add1b.jpeg]

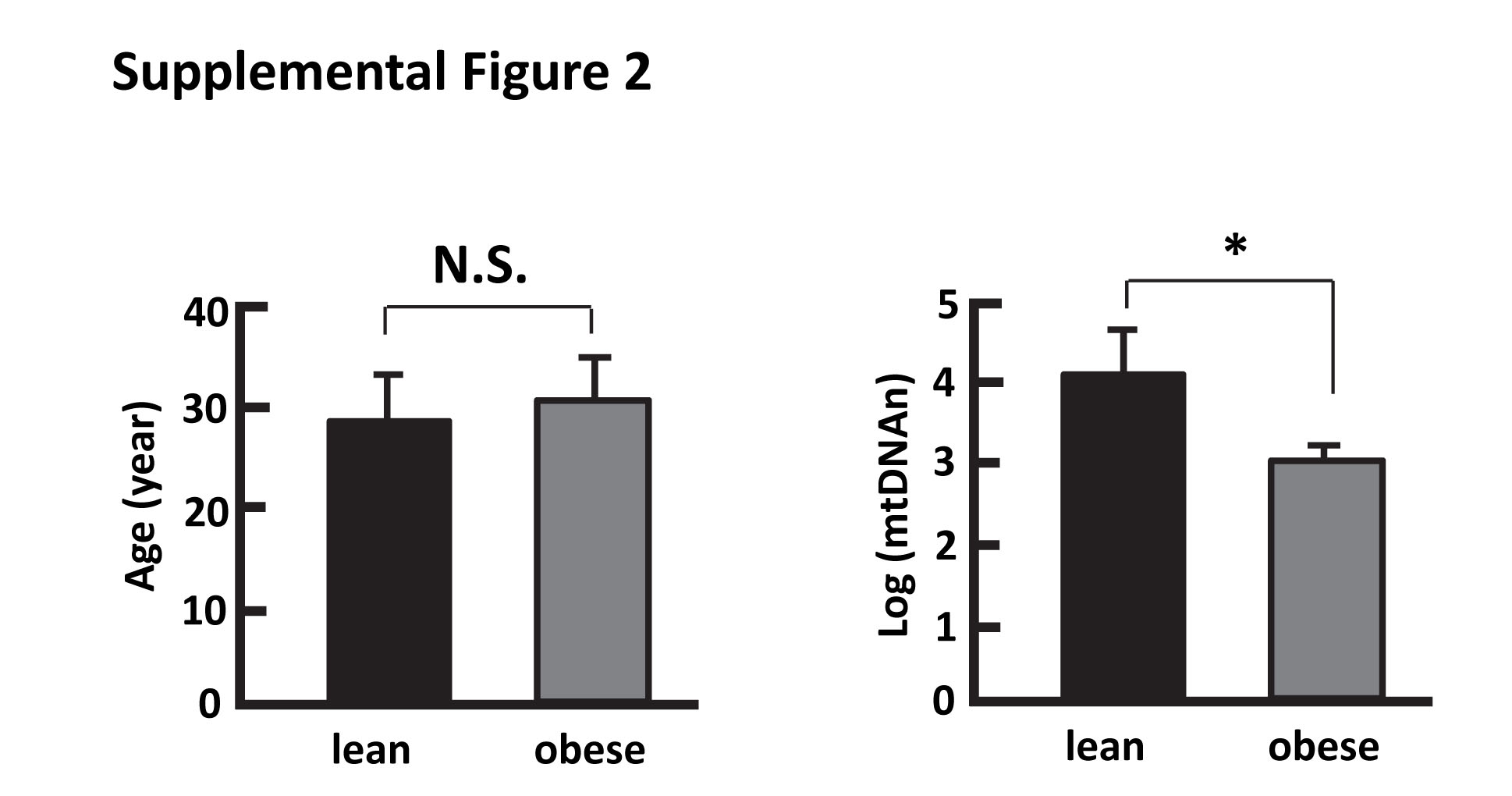

Supplement: Additional file 2: Figure S2. — Age-matched analysis of mtDNAn in lean (n = 7) and obese (n = 8) participants. (A) No significant difference existed between the ages of lean (n = 7) and obese (n = 8) participants. (B) Comparison of mtDNAn between lean (n = 7) and obese (n = 8) participants. The data were presented as mean ± SE. *p < 0.05; NS, not significant. [file 13148_2015_93_MOESM2_ESM.jpeg]

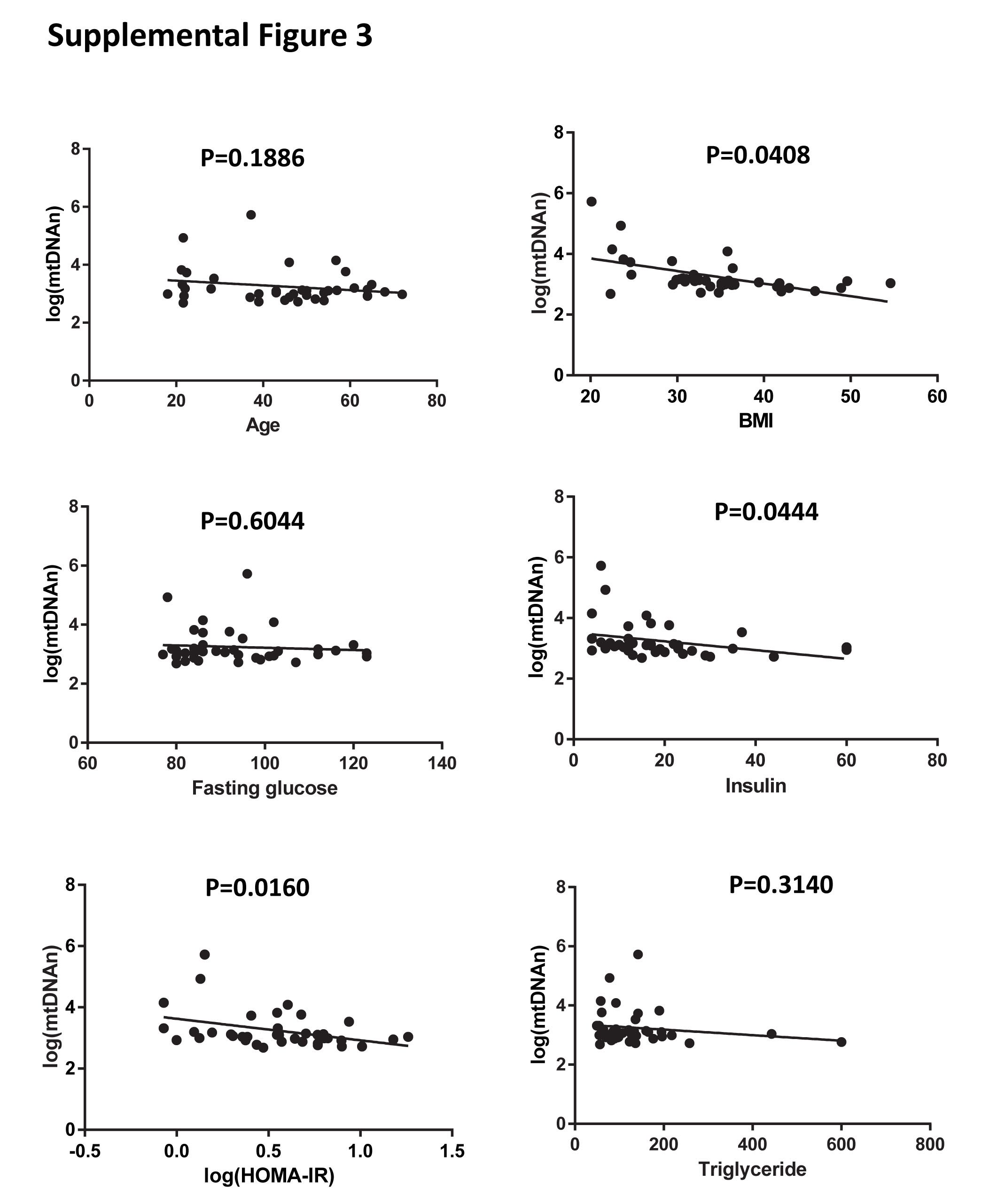

Supplement: Additional file 3: Figure S3. — Regression analyses of mtDNAn with metabolic parameters and demographic characteristics (n = 40). These graphs correspond to the analysis and data shown in Table 2. [file 13148_2015_93_MOESM3_ESM.zip › 13148_2015_93_add3a.jpeg]

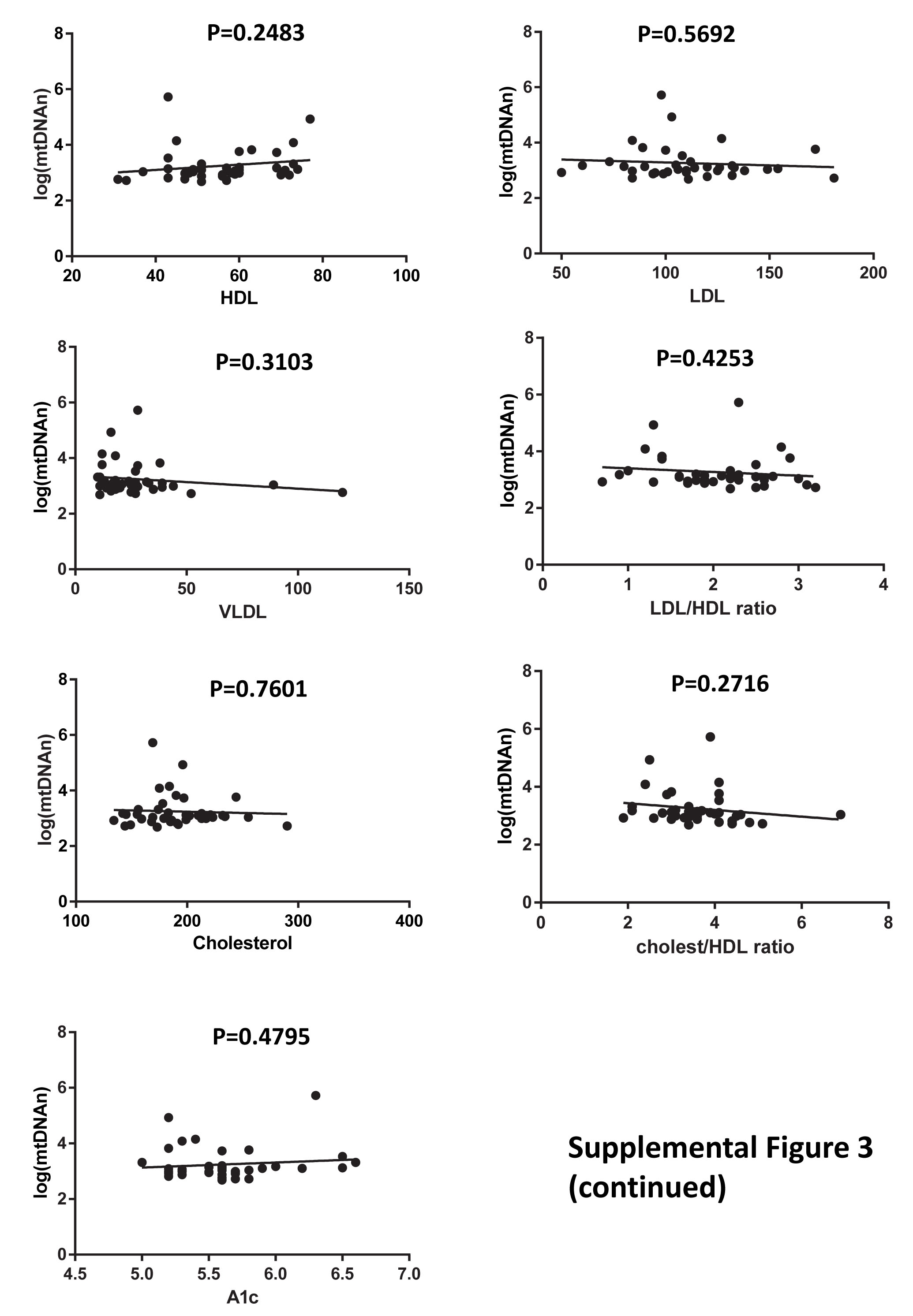

Supplement: Additional file 3: Figure S3. — Regression analyses of mtDNAn with metabolic parameters and demographic characteristics (n = 40). These graphs correspond to the analysis and data shown in Table 2. [file 13148_2015_93_MOESM3_ESM.zip › 13148_2015_93_add3b.jpeg]
